# Managing an Older Adult with Cancer: Considerations for Radiation Oncologists

**DOI:** 10.1155/2017/1695101

**Published:** 2017-12-13

**Authors:** Sanders Chang, Nathan E. Goldstein, Kavita V. Dharmarajan

**Affiliations:** ^1^Icahn School of Medicine at Mount Sinai, New York, NY, USA; ^2^Brookdale Department of Geriatrics and Palliative Medicine, Mount Sinai Hospital, New York, NY, USA; ^3^Department of Radiation Oncology, Mount Sinai Hospital, New York, NY, USA

## Abstract

Older adults with cancer present a unique set of management complexities for oncologists and radiation oncologists. Prognosis and resilience to cancer treatments are notably dependent on the presence or risk of “geriatric syndromes,” in addition to cancer stage and histology. Recognition, proper evaluation, and management of these conditions in conjunction with management of the cancer itself are critical and can be accomplished by utilization of various geriatric assessment tools. Here we review principles of the geriatric assessment, common geriatric syndromes, and application of these concepts to multidisciplinary oncologic treatment. Older patients may experience toxicities related to treatments that impact treatment effectiveness, quality of life, treatment-related mortality, and treatment compliance. Treatment-related burdens from radiotherapy are increasingly important considerations and include procedural demands, travel, costs, and temporary or permanent loss of functional independence. An overall approach to delivering radiotherapy to an older cancer patient requires a comprehensive assessment of both physical and nonphysical factors that may impact treatment outcome. Patient and family-centered communication is also an important part of developing a shared understanding of illness and reasonable expectations of treatment.

## 1. Introduction

*Vignette*. Robert Smith is a 75-year-old functionally independent gentleman with recently diagnosed Stage II laryngeal carcinoma. His radiation oncologist prescribed him a schedule of 70 Gy in 2 Gy fractions [1]. Mr. Smith tolerated the initial weeks of radiation therapy well; however, by week 4, he developed moderate mucositis and his oral intake significantly decreased. This led to subsequent dehydration, resulting in an episode of syncope that caused him to fall down a flight of stairs. Mr. Smith was hospitalized with a fractured hip and underwent surgery to stabilize the fractured bone. He required a 2-month stay in a nursing facility due to loss of his functional independence.

This above vignette is an unfortunately common scenario that could complicate the cancer treatment process for an older patient. An event such as Mr. Smith's fall may at first seem to be an ill-timed coincidence unrelated to cancer treatment. In reality, it is a consequence of his complex multifaceted health and functional status. Many older adults risk a predicament similar to Mr. Smith's in the face of cancer treatment. As radiation oncologists, how can we better conceptualize assessment and management for patients like Mr. Smith and reduce the risk of events leading to a loss of functional independence?

Acknowledgment of older adults with cancer as a distinct patient population requiring knowledge of specialized principles in cancer management is increasingly important given that the population of the United States is aging at an increasing rate. A projected 74.1 million (20.6%) of Americans will be 65 or older in 2030, an increase from 46.2 million (14.5%) in 2014; 9.1 million (2.5%) of Americans will be 85 or older in 2030, an increase from 6.2 million (1.9%) in 2014 [2]. Given the linear relationship between increasing age and cancer risk, the number of older adults with cancer will increase dramatically over the next decade. Between 2010 and 2030, an anticipated increase of 67% will be expected in the cancer incidence for patients 65 years or older (1.0 million to 1.6 million instances) [3]. A significant proportion of these patients will receive radiation therapy as part of their treatment. It has been projected that from 2010 to 2020, there will be a 38% rise in the number of adults 65 or older who will be treated with radiation therapy as an initial treatment course (282,000 to 388,000 instances) [4].

The key to approaching management of older patients with cancer is to understand the unique differences that these patients have compared to their younger counterparts, both with respect to their tumor biology and overall health and functional status. Older patients have distinct physical, psychosocial, and economic needs that play an important part in their well-being. In order to comprehensively address their care, radiation oncologists may find it helpful to have a working understanding of the available tools for performing a geriatric assessment and the common geriatric syndromes such as falls, frailty, and polypharmacy that might affect treatment outcomes of older adults. A common pitfall that arises in oncologic management of older adults is making decisions about medical management based primarily on the type and stage of cancer and chronological age. However, decision-making in this special population is significantly more nuanced. Assessment of* functional age* [5], which is distinct from chronological age and is determined by a combination of factors including performance status, comorbidities, and presence of geriatric syndromes, is of paramount importance. This article will review the utility of geriatric assessment tools that may help predict a patient's ability to tolerate cancer treatments and side effects, the geriatric syndromes that can come into play during cancer treatment, and the specific considerations regarding the delivery of radiation therapy and chemotherapy to older adults.

## 2. Phenotype of the Older Adult

Aging is a coordinated process associated with many physiological and biologic changes in the human body. Over time, organs gradually lose their maximal function and respond less resiliently to external stresses [6]. At the cellular level, senescence may lead to reduced replication efficiency and recovery of tissues. At the molecular level, a lifetime of DNA replication, damage, and repair can pave the way to deleterious mutations that can ultimately lead to cancer. These changes play a part in the pathological development of morbidity and mortality in the older patient as well as overall resiliency and recovery.

### 2.1. Morbidity in the Older Adult

With a growing population of older adults, morbidity is becoming an increasing concern in the geriatric population. Compared to their younger counterparts, older adults harbor a greater number of chronic conditions. Many suffer from syndromes unique to the geriatric population such as frailty, dementia, and falls and can rely increasingly upon caregiving. From a 2014 report on people aged 65 years or older residing in the United States, 55.9% were living with hypertension, 49.0% with arthritis, 29.4% with heart disease, 23.4% with cancer, and 20.8% with diabetes [2]. More than 50% of Medicare beneficiaries have more than one chronic condition [7]. Increasing level of multimorbidity is positively correlated with increasing number of inpatient admissions, postacute care services, home health visits, emergency room visits, and readmissions to the hospital within 30 days of discharge [7]. This overall decrease in health status of older adults results in a higher utilization of health care services. On average, older patients incur more annual health care costs, which consist of hospital stays, doctors' visits, nursing home or visiting nurse services, and prescription drugs [2]; yet at the same time, they may not have steady financial resources to pay for these increasing costs, some of which may be out-of-pocket expenses.

## 3. Geriatric Assessment

### 3.1. Principles of the Geriatric Assessment

An older person's ability to perform activities of daily living may impact the tolerability of cancer treatments. An awareness of the multidimensional contributors to the overall health status of an older adult (separate from the cancer stage, Karnofsky Performance Status at the time of visit, and an individual's chronological age) may allow for conceptualization of more optimally tailored treatment recommendations for an individual patient and also may better prepare the patient and caregivers for what to expect during treatment and afterwards. A full assessment of the geriatric patient covers each of the following domains: physical condition, cognitive function, functional status, nutritional status, psychosocial health, economic status, physical environment, caregiver support, and spirituality [6]. Specific issues that might have important implications during a patient's treatment and thus need to be considered during an oncologic geriatric assessment include but are not limited to impairments of vision and hearing, urinary incontinence, constipation, changes in gait, history of falls, tremor, neurocognitive deficits, and changes in weight.

### 3.2. Tools for the Geriatric Assessment

The “gold standard” for evaluating older adults is the comprehensive geriatric assessment (CGA). The origin of the CGA dates back to the 1940s by Dr. Marjory Warren in the United Kingdom, who noticed a need to better manage older patients in the hospital who were bedridden and chronically ill [49]. She developed one of the first geriatric units to help mobilize these patients to undergo proper medical and rehabilitative care, thereby paving the way for a more systemic means of evaluating geriatric patients. A CGA involves a methodical approach of in-depth evaluation that includes an assessment of four health domains: physical health, functional status, psychological well-being, and socioeconomic factors [6]. Many screening tools and questionnaires can be used to assess these different health domains (summarized in Table 1), although not all of them are necessarily required to perform a thorough CGA.

The use of such a systematic way to conduct a full geriatric assessment has utility in predicting survival outcomes among older patients undergoing cancer treatment [50]. For instance, better-performing older adults had improved survival following surgery for breast or colorectal cancers, whereas poorer-performing older adults had a higher postoperative mortality risk [50, 51]. Risk-stratifying patients based on a CGA may uncover certain parameters (e.g., low vitamin D levels) that could be intervened upon and thereby minimize treatment-related toxicities [52, 53]. Evidence has shown that performing a CGA can alter up to 49% of patients' initial treatment plans [54]. In radiation therapy, conducting a CGA has the potential to predict side effects and tolerability to radiation. A recent Turkish study found that certain parameters measured in the CGA, such as low vitamin D levels and slower 6-meter length gait speeds, are associated with postradiation esophagitis and emesis, respectively [53].

Performing a CGA can be time consuming and resource intensive, and the use of these tools may require additional training. Several abbreviated screening tools have been proposed which identify patients who would likely benefit from a full CGA (Table 2). Each of the tools has undergone assessment of its sensitivity and specificity [41]. For example, the G8 had a sensitivity ranging from 65% to 92% and specificity ranging from 3% to 75%. VES-13 had a sensitivity ranging from 39% to 88% and specificity ranging from 62% to 100%. Because of their varying rates of sensitivity and specificity, there is ongoing debate on whether these screening tools can be used alone or in conjunction with a full geriatric assessment [41, 55]. Despite its wide variation in sensitivity range, direct comparisons between G8 and other screening tools show that G8 performs at a significantly greater or equal sensitivity [41, 43]. G8 may be the preferred screening tool in a radiation oncology setting, given its performance and efficiency.

The European Organisation for Research and Treatment of Cancer QLQ-C30 (EORTC QLQ-C30) is another widely used tool that can assess the quality of life for cancer patients undergoing treatment but is nonspecific for older adults [56]. An updated version of this tool (EORTC QLQ-ELD14) validated for older patients has been in use since 2013 but is not specifically designed to assess for geriatric syndromes [57].

## 4. Geriatric Syndromes

An explanation for the increased morbidity and mortality of older patients lies in their susceptibility to a set of conditions known as “geriatric syndromes.” These ailments are a central concern in geriatric care and include frailty, dementia, syncope, delirium, falls, dizziness, sleep disorders, and pressure ulcers [6]. Frailty is a well-studied geriatric syndrome considered highly relevant in cancer treatment and specifically in radiation therapy.

### 4.1. Frailty

As patients age, changes in physical health and functional abilities become increasingly complex and thereby cannot be easily attributed to a single underlying clinical condition (such as cancer). A unifying factor that can explain these multifaceted changes is the concept of frailty, which is defined by geriatricians as a vulnerable, age-related state in which one is less able to maintain homeostatic equilibrium, resulting in unfavorable outcomes such as falls and disability [6, 18, 58, 59]. Phenotypically, the frail patient will exhibit a range of manifestations, such as loss in energy, physical strength, and weight, and inability to perform common functional tasks. In recent years, frailty is appreciated more broadly as a multidimensional concept of illness spanning biological, physiological, psychological, and social domains of the older adult [60–62].

The prevalence of frailty in the geriatric population is unclear. A systematic review by Collard et al. of populations in various countries (e.g., United States, Canada, and United Kingdom) found the prevalence of frailty to be 4.0% to 59.1% among community-dwelling adults 65 or older [63]. This wide range is attributed to the observation that a proportion of the studies defined frailty solely as a physical phenotype, which had a weighted prevalence of 9.9%, whereas another proportion defined it as both a physical and psychological phenomenon, which resulted in a weighted prevalence of 13.6%.

Several metrics have been proposed to quantify the degree of frailty in a patient [64]. The two most prominent scales are the CGA-based Frailty Index (FI), which assesses the accumulation of deficits in various areas spanning physical symptoms, psychological symptoms, functional abilities, and behaviors [16, 17], and the Fried Frailty Index (FFI), which addresses frailty as solely a physical phenotype (Table 3) [18]. Other scales, like the Groningen Frailty Indicator (GFI) and the Tilburg Frailty Indicator (TFI), are variants of these two approaches [47, 65]. Generally, not one tool is preferred over another, as each evaluates complementary aspects of the health and functional status of geriatric patients, which explains the wide prevalence of frailty in the systematic review by Collard et al. [63, 64, 66].

Assessing frailty has importance in preparing patients for cancer treatments and radiotherapy. Frail patients are more likely than nonfrail patients to exhibit more side effects or have complications from both chemotherapeutic drugs and radiotherapy treatments and at the same time have less functional capacity with which to overcome these adverse effects. From one systematic review, it has been calculated that the median estimates of frailty and prefrailty in older patients with cancer are 42% (range 6%–86%) and 43% (range 13%–79%), respectively [67]. Frailty was found to increase all-cause mortality, postoperative mortality, postoperative complications, and treatment toxicities from procedural and/or chemotherapeutic interventions [67].

For a radiation oncologist, the construct of frailty in older patients can be considered a measure of functional reserve. In this context, patients identified as frail may be less likely to recover from radiation treatments compared to their younger counterparts. The radiation oncologist can consider utilizing frailty to help better evaluate the older patient when planning their treatments. In a sample of older women with breast cancer, 26% had a baseline Fried frailty score > 1 [68]. Radiotherapy-related fatigue was better predicted by the Fried frailty score than other assessments such as the Karnofsky Performance Status. A higher score on a Frailty Index modified to include the presence of comorbidities like diabetes and history of stroke was associated with significantly lower overall survival and other cause-specific survival in stage I/II non-small cell lung cancer patients who underwent stereotactic body radiation therapy (SBRT) [69].

Understanding the risks and toxicities associated with frailty during cancer treatments may help the older patient withstand and recover from the effects of chemotherapy and radiotherapy [70]. Frailty is a formal construct in geriatrics. In oncology, an analogous concept is that of functional reserve. Management of frailty relies first on its proper diagnosis and second on an understanding of how expected toxicities arising from cancer treatment might precipitate an event leading to an adverse outcome in a frail patient such as a fall (as in the case of the patient described in the above vignette). Several management options exist for health conditions and treatment complications that might result or get worse from frailty existing at baseline. These include pharmacologic interventions for sarcopenia, cachexia, and nutritional deficits [71–73] and exercise interventions [74]. Overall, management should be multidisciplinary and involve physical therapists, dieticians, nurses, caregivers, geriatricians, oncologists, and radiation oncologists [75]. Understanding how frailty plays a role in the care of the older cancer patient allows us to more thoughtfully address how certain reduced capabilities might affect a person's tolerability of current cancer treatments.

## 5. Special Radiation Treatment Considerations

Radiotherapy is an important anticancer modality and sometimes the treatment of choice for patients regardless of age or comorbid condition (for example, prostate cancer). A decision to pursue radiotherapy requires a full comprehensive understanding of the goals of treatment, individual patient characteristics, and the predicted tolerability to the radiation treatment itself. An incomplete understanding of a patient's upfront ability to tolerate the effects of treatment may lead to interruption of treatment courses, which can lead to undesirable cancer repopulation [76, 77]. Clinicians may need to be extra careful of issues or problems that might arise during radiation treatment in the older individual. These include toxicities from radiation, treatment-related burdens persisting after radiation, and synergistically related toxicities resulting from combined chemoradiation.

### 5.1. Issues regarding the Biology of the Older Human Body

Older adults are susceptible to the same toxicities of radiation that affect their younger counterparts [78]. In general, radiation can cause irreversible damage to cells and tissues in the human body, resulting in acute injury manifesting a few weeks after radiation treatment, or long-term reduction in organ function manifesting months or years after [78]. Older adults, given their likelihood of having organs worse in function, are presumably more vulnerable to the toxicities of radiation therapy. Interestingly, in vitro studies have not shown whether age has any impact on the radiosensitivity of primary human cancers cells like fibroblasts, breast cancer cells, mucosa, and vascular smooth cells [79–82]. Clinically, there is evidence of worsening functional impairment in older patients treated with radiotherapy [78]. Some of the most concerning toxicities that older adults may have a higher vulnerability of experiencing are fatigue [83, 84], mucositis, xerostomia, dehydration, infections [78], and cognitive defects [85–88]. However, other studies have produced contradictory results regarding this subject [89–91]. There is continued controversy on whether a correlation exists between chronological age and incidence of radiation-related toxicities.

### 5.2. Increased Treatment Precision Can Reduce Toxicities

Over the years, advances in technology have led to radiation techniques that efficiently deliver adequate amount of radiation to an area with potential for reduced toxicity. Intensity-modulated radiation therapy (IMRT) and image-guided radiation treatment (IGRT) techniques allow the delivery of higher doses in the treatment volumes with better sparing of surrounding normal tissue [92]. These techniques have been associated with lower toxicities than other forms of radiation therapy in various types of cancers including cancer of the prostate, head and neck, breast, bladder, and rectum [93–97]. Stereotactic body radiation therapy (SBRT) allows for higher dosages to be delivered to a targeted tumor region with fewer numbers of fractions [98] and has been associated with a lower incidence of toxicities [99], such as radiation pneumonitis in NSCLC treatment for instance [100]. A recent study on brain metastases found that stereotactic radiosurgery (SRS) alone compared with SRS with whole brain RT can lead to a better conservation of cognitive ability and quality of life without compromising survival [101]. The advent of more precise radiation technologies has presented safer and efficient options of radiation that can be delivered to older patients with better toxicity profiles [94, 102–104].

Despite recent advances in precision technology, the functional status of older adults with physical impairments and/or comorbidities remains of utmost importance when considering the appropriate radiation treatment. Frailty can be a predictor of radiation-induced toxicity in older patients [68, 69]. The presence of competing noncancer risk factors can negatively impact tolerance to radiotherapy. Women with breast cancer who also have a comorbid heart condition can be at higher risk of developing cardiotoxicity to radiotherapy treated to the breast [105].

Given their likelihood of experiencing increased toxicities, older adults are at risk of not being able to complete extended radiation treatments because of their amplified experience of side effects, decline in clinical status, or treatment burden regarding transport and financial costs [106, 107]. These patients would thereby benefit from hypofractionated treatment schedules, which deliver the same total doses as that of conventional schedules, but in a smaller number of treatment sessions (fewer visits to the treatment facility of hospital). There is also growing evidence that geriatric assessments may help with risk-stratifying patients receiving radiotherapy. One study found that a Vulnerable Elders Survey-13 (VES-13) score above 7 was associated with around 3 times greater probability for not completing a radiation treatment [108]. A prognostic index consisting of demographics, medication and hormone use, comorbidities, anxiety, and functional status was validated for patients with prostate cancer who underwent androgen deprivation therapy and/or radiation therapy [109]. Patients determined to be high-risk by the prognostic index had a significantly higher risk for fracture rate (19.2% in derivation cohort, 16.5% in validation cohort).

### 5.3. Issues regarding Treatment-Related Burdens

Radiation treatments can negatively impact the quality of life for older adults in ways other than acute and long-term toxicities. As described above, older patients can be encumbered by geriatric syndromes that can reduce the effectiveness of anticancer treatments. Protocols and fractionation schedules that do not acknowledge the severity of these syndromes can subject older patients to treatment noncompliance and further deterioration in health.

The efficacy of radiation treatment lies in its successful delivery. To this end, patient cooperation throughout radiation treatment procedures is necessary. Presence of certain geriatric syndromes might affect a patient's ability to complete parts of the radiation treatment process [6]. For instance, patients with hearing impairments, highly prevalent among older adults, may not be able to promptly listen to directions during dynamic (e.g., respiratory motion management) protocols. Similarly, patients with dementia may not be able to verbalize sensations of discomfort or pain during radiation or remember instructions. Furthermore, patients with movement disorders like Parkinsonian tremors, or severe arthritis, may have difficulty with immobilization or positioning. Those with frailty or severe physical impairment may have difficulty accomplishing stressful maneuvers such as breath-holding or abdominal compression [102]. A focused evaluation of these potential issues upfront may allow workaround solutions to be developed that could make treatments less taxing and more manageable. Special accommodations may be made by treatment facilities or hospitals to make these techniques possible for patients with certain disabilities. For instance, respiratory motion management techniques can have visual or auditory guides (e.g., screens inside treatment rooms, special glasses, and sound alarms).

Over the past decade, newer approaches have been developed that can potentially address some of these technical challenges. Respiratory motion tracking allows for radiation to be delivered without the need for a breath hold [110]. However, these techniques tend to lengthen the treatment session time, which can increase patient discomfort (common in older patients, as previously mentioned). Four-dimensional computed tomography (4D-CT) and image guidance (4D-IG) tracks organ movement over time through frequent image capture during the radiation course [111, 112]. Adaptive radiation therapy (ART) individualizes radiation treatment by replanning and redosing radiation daily, allowing for complicated patients who require varying treatment set-ups [113, 114].

Another important consideration must be made for the socioeconomic factors that often play a role in radiotherapy effectiveness in older adults. Extended fractionation schedules may require frequent travel between the radiotherapy facility and the patient's residence. Since older adults are vulnerable to treatment-related fatigue and deficits in physical activity, constant travel may severely impact their quality of life or may simply not be feasible [115–117].

In consideration of the socioeconomic factors and toxicities, older patients may fare better with shorter fractionation schedules without compromising tumor control. Hypofractionation has been studied to be an effective alternative to conservative fractionation in different cancers [118–120]. In breast cancer, older patients treated with hypofractionated RT (42.56 Gy in 16 fractions) as opposed to standard RT (50–60 Gy in 25–30 fractions) had 100% overall survival and no severe toxicities or local recurrence at median follow-up of 15 months [120]. In glioblastoma multiforme (GBM), patients over 60 years of age receiving 40 Gy in 15 fractions over 3 weeks had similar overall survival to those who were treated with the standard 60 Gy course [121]. In head and neck cancer, patients with multiple comorbidities and overall poorer health may be able to tolerate split-course, accelerated, hypofractionated radiotherapy (SCAHRT), or a regimen comprising 60–72 Gy in 20–24 fractions with a break in the middle of the course [119].

Hypofractionated courses can also be useful in the palliative setting. For instance, in palliation of bone metastases, a lower incidence of acute toxicities (e.g., nausea and vomiting, diarrhea, and fatigue) was observed in 8 Gy in 1 fraction compared to 30 Gy in 10 fractions in some randomized trials [122].

However, hypofractionated courses may present with an additional inconvenience for older patients. Although the physical dose is lower, the dose per session is higher, which may lead to a higher likelihood of acute toxicities in normal tissues if the same tolerances used in normofractionated schedules are applied. It is important to emphasize the necessity of adjusting the dose constraints in hypofractionated schedules.

A reasonable alternative to delivering radiotherapy is omitting it in favor of more supportive measures. Supportive care alone may benefit some selected cancer patients [123]. However, caution must be made before making the decision to omit radiotherapy, as withholding adjuvant radiotherapy can risk tumor recurrence and worsening of tumor progression [124, 125].

### 5.4. Issues regarding Concurrent Chemoradiation Treatments

It has been shown that combined chemoradiation can improve survival in certain cancers, like that of head and neck, brain, endometrium, and lung [126–129]. However, chemotherapy adds toxicities that can compound those of radiation therapy, such as mucositis, cytopenia, and cardiotoxicity [130, 131]. In addition, because of their age-related reductions in kidney and liver function, older adults are prone to increased chemotherapy potency [6]. Evidence has shown that older individuals receiving combined chemoradiation treatments can experience amplified toxicities leading to more frequent hospitalizations and worse survival [132].

On the other hand, some trials have revealed that older patients may be able to tolerate chemoradiation for particular cancers similar to younger counterparts [133, 134]. However, most of these trials only included older individuals who were medically fit and with few or no comorbidities [135]. Thus, results may not be routinely generalizable to patients with an increased number of comorbidities or functional impairments.

Toxicities may be better controlled by employing the use of more precise radiation technologies during chemoradiation. Chemoradiotherapy using IMRT for treatment of cervical cancer has been shown to limit spread of radiation to the bone marrow and reduce incidence of hematologic toxicity [136]. Use of tomotherapy-based IGRT in chemoradiation for small cell lung cancer was associated with no grades 3 to 4 pneumonitis, although other toxicities like esophagitis and pulmonary embolism was still observed in some patients [137].

Combining chemotherapy with hypofractionated radiation may be feasible without increasing overall toxicity. A retrospective analysis examining the concurrent use of temozolomide (TMZ) and radiotherapy in glioblastoma found that older patients receiving hypofractionation and TMZ generally tolerated the combined regimen well [138].

Older adults with poorer functional status may better tolerate sequential, rather than concurrent, chemoradiation [139]. One study found that patients with nasopharyngeal carcinoma receiving sequential chemoradiation had overall less severe acute toxicities (leukopenia, anemia, mucositis, and weight loss) than those who underwent concurrent chemoradiotherapy; however, there was no significant difference in survival between the two modalities [140]. The use of more precise radiation technologies, hypofractionation, and sequential chemoradiation may benefit older individuals with poor functional status.

## 6. A Framework for Making Management Decisions

A central question is how to best utilize the skillset of specialty-level geriatrics to optimize cancer treatment for older patients. A conceptual model utilizing four domains (tumor behavior, noncancer related competing risks, functional reserve, and palliative needs) described by G. L. Smith and B. D. Smith can be considered a foundation for making tailored radiation treatment plans [141]. A comprehensive geriatric assessment and other abbreviated, easy-to-use geriatric assessment screening tools augment this model by facilitating ascertainment of objective data about competing health risks and functional reserve [8, 54]. Routine use of these assessment tools in practice may help risk-stratify geriatric patients and guide treatment decision-making. An objective and complete assessment of an older patient may uncover potentially modifiable geriatric impairments. These in turn might affect the choice of radiation treatment modality, set-up, technique, dose, and fractionation that would be most appropriate based upon an individual's unique set of clinical characteristics and circumstances [50, 51, 53, 67]. At the same time, a full geriatric assessment may be time-consuming and resource intensive [41]. Not every cancer clinician may be trained to perform one, and not every older individual may require one. Additional studies are needed to find reliable, routine, and easy-to-use screening tools for radiation oncologists' use that can expedite assessment [41, 142].

Choosing the “best” radiotherapy plan for the older adult requires clinical judgment and acknowledgment of patient preferences and concerns. Quality of life and functional independence may be highly valued among older adults with cancer. At the same time, undergoing radiotherapy, like any other anticancer treatments, can be an arduous endeavor and may be associated with temporary or permanent detriments in quality of life and function that persist and even worsen at times after treatment is over. An upfront discussion of the potential risks, benefits, and acceptable trade-offs of treatment can be more thoroughly and clearly conducted by recognizing the multidimensional factors in caring for the older adult and screening for geriatric impairments that might directly impact a patient's treatment outcome. It may be reasonable in many instances to involve extra supportive services earlier in a treatment course for older adults at higher risk (e.g., significant comorbidities and frailty, or for whom cancer treatments may be particularly burdensome). These might include supportive oncology specialists whose role is to add a layer of support for both patients and caregivers. This type of care may be delivered by palliative care clinicians whose role is to care for any type of cancer patient regardless of cancer type, stage, intent of treatment, or age. Incorporating supportive oncology specialists earlier in the management of cancer patients is associated with better patient-reported quality of life in multiple domains [143].

It is important for radiation oncologists to recognize the limitations that their older patients may have in terms of completing radiation treatment courses. Many patients may experience treatment interruptions for a variety of reasons (toxicity-related, patient-related, caregiver-related, treatment machine-related, etc.). The efficacy of the treatment, however, depends upon it being completed with minimal interruptions [76, 77]. Thus, radiation oncologists may keep this in mind as they make recommendations during consultation and counsel their patients during on-treatment visits in order to prevent and/or reduce these interruptions as much as possible. By bringing specific mention to the potentially harmful effects that many interruptions can cause, radiation oncologists and patients/caregivers can decide together what treatment regimens are most appropriate.

The data gathered from a geriatric assessment allows radiation oncologists to deepen their understanding of potential treatment implications for older patients in a way that can facilitate better informed shared decision-making. A multidisciplinary supportive care approach involving geriatric expertise, social work services, visiting nurse assistance, nutritional support, physical therapy, and others can be employed in a timely manner, possibly preventing a consequence of treatment such as that described in the introductory vignette. Supportive services may also include individuals specializing in psychosocial oncology [144]. Attention to psychosocial health is a critical aspect of comprehensive supportive care for cancer patients of all ages.

## 7. Conclusion

The overall approach to delivering any cancer treatment for the geriatric patient, whether it consists of surgery, chemotherapy, radiotherapy, or the combination of these, requires a global understanding of physical, functional, and social well-being. Assessment tools are available for more optimal evaluation of older individuals with cancer. Integrating abbreviated versions of these tools is feasible to do within the routine flow of a radiation oncology clinic. The decision to pursue specific treatments requires patient-centered communication of preferences, concerns, risks, and benefits among patients, caregivers, and clinicians.

## Figures and Tables

**Table 1:** Examples of available screening tools currently used to conduct a geriatric assessment (CGA), adapted from [8, 9].

General health status domain	Specific domain components	Screening tools available for assessment of the specific domain components
Physical health status	Comorbidities	Charlson Comorbidity Index (CCI) [10], Cumulative Illness Rating Scale for Geriatrics (CIRS-G) [11]
	Nutrition	Subjective Global Assessment (SGA) [12], Mininutritional Assessment (MNA) [13], Geriatric Nutritional Risk Index (GNRI) [14]
	Medications	Review history of medications, Beers criteria [15]

Functional status	Frailty	Frailty Index (FI) by deficit accumulation [16, 17], Fried Frailty Index [18], Vulnerable Elders Scale-13 (VES-13) [19]
	Activities of daily living (ADLs)	Barthel's Index Rating Scale [20], Katz Index of Independence in ADLs [21]
	Instrumental activities of daily living (IADLs)	Functional Activity Questionnaire [22], Rapid Disability Rating Scales [23]
	Falls and balance test	History of falls, Berg Balance Scale [24], Timed Up and Go Test [25], Tinetti Gait and Balance Test [26], Fall Risk Assessment Scale for the Elderly (FRASE) [27], Fall Risk Index [28]
	Gait speed	Average In-home Gait Speed (AIGS) [29]
	Strength	Handgrip Test [30]

Psychological well-being	Cognitive function	Minimental Status Examination (MMSE) [31], Montreal Cognitive Assessment (MoCA) [32], Informant Questionnaire on Cognitive Decline in the Elderly (IQCODE) [33], Simple Clock Drawing Test
	Depression and anxiety	Geriatric Depression Scale (GDS) [34, 35], Hamilton Rating Scale for Depression (HRSD) [36], Geriatric Anxiety Inventory (GAI) [37], Geriatric Anxiety Scale [38], Hospital Anxiety and Depression Scale [39]

Socioeconomic status	Social support	General questionnaire, Medical Outcomes Survey Social Support [40]
	Environment	Financial capabilities, transport facilities, technology use, home safety questionnaires

**Table 2:** Selected screening tools currently available to perform an abbreviated geriatric assessment, adapted from [41].

Screening tools	Purpose	Method of assessment	References
G8 screening questionnaire	Identify geriatric impairments in elderly patients across all CGA domains	8-item clinical assessment conducted by health care provider: food intake, weight loss, mobility, neuropsychological problems, body mass index, medication usage, self-perception of health, and age	[42]

Vulnerable elders survey-13	Identify elderly patients who are “vulnerable,” that is, at risk of functional worsening or death over 2 years	12-item clinical assessment conducted by health care provider: physical activities, ADL/IADLS, age, self-rated health, and comorbidities	[19]

Flemish version of the triage risk screening tool	Identify elderly patients who are at risk for readmission following discharge	5-item clinical assessment conducted by health care provider: presence of cognitive impairment, living alone or no caregiver available, walking difficulty and history of falls, recent hospitalization, and polypharmacy (≥5 medications)	[43, 44]

Study of osteoporotic fractures index	Measure “prefrailty” and “frailty”	3-item clinical assessment conducted by health care provider: weight loss, inability to rise from chair, and poor energy	[45, 46]

Groningen frailty indicator	Measure physical, social, and/or psychological impairment	15-item clinical assessment conducted by health care provider: mobility, vision, hearing, nutrition, comorbidities, cognition, psychosocial, and physical fitness	[47]

Fried frailty criteria	Measure “frailty”	5-item clinical assessment conducted by health care provider: weight loss, handgrip, gait speed, exhaustion, and physical performance	[18]

Abbreviated comprehensive geriatric assessment (aCGA)	Select items from the CGA to expedite assessment	15-item clinical assessment conducted by health care provider: from Geriatric Depression Scale [34], MMSE [31], ADLs, and IADLs	[48]

**Table 3:** Phenotypic criteria for the Fried Frailty Index, adapted from [18].

Characteristics of frailty	Criteria used to define frailty (from Fried et al.)
Shrinking: weight loss (unintentional), sarcopenia (loss of muscle mass)	Baseline: >10 lbs lost unintentionally in prior year

Weakness	Grip strength: lowest 20% (by gender, body mass index)

Poor endurance; exhaustion	“Exhaustion” (self-reported)

Slowness	Walking time/15 feet: slowest 20% (by gender, height)

Low activity	Kcals/week: lowest 20% (i) Males: <383 Kcals/week (ii) Females: <270 Kcals/week

*Presence of frailty*	
Positive for frailty phenotype: ≥3 criteria present	
Intermediate or prefrail: 1 or 2 criteria present	
